# Tuberculous Lymphadenitis in a Patient Receiving PD-1 Inhibitor for Melanoma: A Case Report and Brief Literature Review

**DOI:** 10.3390/curroncol28010028

**Published:** 2021-01-04

**Authors:** Baran Akagunduz, Muhammet Ozer, Ali Cagatay Bozkina, Banu Lebe

**Affiliations:** 1 Department of Medical Oncology, Erzincan Binali Yildirim University, 24030 Erzincan, Turkey; drbaran04@hotmail.com; 2 Department of Internal Medicine, Capital Health Regional Medical Center, Trenton, NJ 08638, USA; 3 Department of Internal Medicine, Dokuz Eylul University School of Medicine, 35035 Izmir, Turkey; alicagataybozkina@gmail.com; 4 Department of Pathology, Dokuz Eylul University School of Medicine, 35035 Izmir, Turkey; banu.lebe@deu.edu.tr

**Keywords:** tuberculous lymphadenitis, nivolumab, melanoma, immune checkpoint inhibitors, immunotheraphy

## Abstract

Oncolytic immunotherapy is a novel and promising approach in clinical oncology practice. Currently, immune checkpoint inhibitors (ICIs) are the first-line treatment options for disseminated melanoma. Nivolumab is a well-defined ICI that blocks programmed cell death 1 (PD-1) and mainly increases anti-tumor immunity. The opportunistic infections are not expected with ICI therapies due to their immune reactivation effects. To date, only a few cancer patients have been reported with activated TB during ICI therapy. Here, we presented a young female patient diagnosed with histologically-confirmed tuberculous lymphadenitis while on nivolumab therapy for metastatic melanoma. The current case report represents the first described tuberculous lymphadenitis case related to anti-PD-1 based monoclonal antibody therapy. The mechanism underlying the development of TB with PD-1 inhibitor use has not been illuminated yet. Triggering of excessive inflammatory responses with ICIs therapy is a potential cause. Considering the increased utilization of ICI-based immunotherapies, the TB screening should be considered in all patients before starting PD-1 inhibitor therapy.

## 1. Introduction

Melanoma is a malignancy that primarily derives from melanocytic cells. In the United States, it is the fifth most common cancer in men and women [1]. After the development of immune checkpoint inhibitors (ICIs), cytotoxic chemotherapies have only a limited role in the management of malignant melanoma. ICIs represent a substantial and emerging field of clinical oncology. Currently, ICIs such as the programmed cell death 1 (PD-1) and programmed cell death ligand 1 (PD-L1) inhibitors are the first-line treatment options for disseminated melanoma [2]. ICIs boost the host immune system against malignant cells, and they fortify anti-tumor immune responses by disrupting the inhibitory T-cell signal pathways in the tumor microenvironment [3].

Tuberculosis (TB) is a worldwide public health problem, representing a significant cause of morbidity and mortality [4]. Tuberculous lymphadenitis is the most common form of extrapulmonary TB [5]. Tuberculous lymphadenitis occurs due to hematogenous dissemination. This occurrence can happen either during primary infection or late manifestation of the reactivation of initial lesions. Cultures for mycobacteria are found to be positive in 40–80% of patients, whereas smear microscopy has low sensitivity, with <10% positivity [6]. Diagnosis is confirmed with fine-needle aspiration, core biopsy, or open biopsy.

Nivolumab is a well-defined ICI; it is a fully humanized IgG4 monoclonal antibody that blocks PD-1 and mainly increases the reactivation of anti-tumor immunity [7]. The immune-restorative effects of ICIs can cause immune-related adverse effects (IrAEs). The most common IrAEs of ICIs are fatigue, rash, pruritus, hepatitis, thyroiditis, arthralgia, diarrhea, vitiligo, and pneumonitis [8]. Hypothetically opportunistic infections are not expected side effects of ICIs, due to their immune reactivation effects. Although limited data exist on the development of TB in cancer patients who receive ICIs, interestingly, the diagnosis of TB after ICI use has been recently increasing [9]. In 2016, the first active pulmonary tuberculosis case was reported in a patient in Japan who was using nivolumab for non-small cell lung cancer (NSCLC) [10]. A recent systematic review of the literature detected a total of 16 patients who developed active pulmonary TB after ICI treatment [9]. Currently, there has been no extrapulmonary TB reported in the literature after ICIs use.

In this case report, we describe a 38-year-old Caucasian female patient who was diagnosed with histologically confirmed tuberculous lymphadenitis while on nivolumab therapy for metastatic melanoma. The current case report represents a meaningful addition to the literature given that it is the first described tuberculous lymphadenitis case related to anti-PD-1 based monoclonal antibody therapy.

## 2. Case Presentation

A 38-year-old Caucasian female patient was initially diagnosed with malignant melanoma. At the time of diagnosis, a 2-cm irregularly shaped mole was detected in the left scapular region. Subsequently, the patient underwent surgical excision. Histopathological exam revealed pT3a stage 3b malignant melanoma with the following features: Breslow’s depth of 3 mm, Clark level IV description, and an invasion into the reticular dermis was present. The mitotic rate was 5/mm^2^. One satellite nodule and one sentinel lymph node sample were positive. Other ulcerations, tumor regression, and/or perineural invasion were not identified in the sections examined. Excision of the lesion and axillary dissection was performed. The lymph node specimen sampled from the left posterior axilla was negative. One-year adjuvant nivolumab treatment was planned.

**Figure 1:**
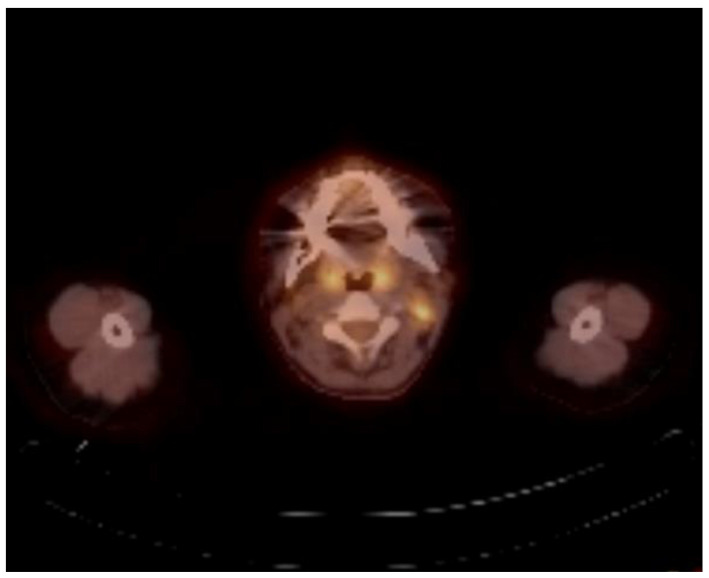
F-18FDG PET/CT imaging shows increased metabolic activity and FDG uptake in left level 2 cervical lymph nodes.

After the fifth cycle of nivolumab treatment, the patient developed a 38 °C fever and grade 2 fatigue. The clinical and biochemical examination did not reveal any immune-related side effects. Physical exams, including skin and the respiratory system, were unremarkable. Thyroid function tests, liver function tests, complete blood count, and basic metabolic panel were within normal limits. We could not find the source of the fever. The patient did not receive any cytotoxic chemotherapy or steroids. During the treatment, the patient did not have any findings suggesting a clinical progression. Upon completion of the sixth cycle, control F-18 fluorodeoxyglucose (F-18FDG) PET/CT detected a significant metabolic progression in the left cervical region lymph nodes (Figure 1).

**Figure 2:**
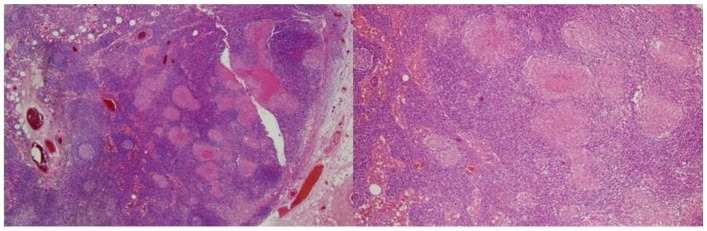
The histopathologic evaluation of the left cervical modified radical type 3 dissection material showing caseous granulomatous lymphadenitis.

Left cervical modified radical type 3 dissection was performed. Meanwhile, the patient’s fever was persistent. The histopathological evaluation of the eight lymph nodes revealed caseous granulomatous lymphadenitis (Figure 2).

Ziehl–Neelsen staining of the material was negative. High resolution computed tomography (HRCT) of the lungs did not show any signs of pulmonary tuberculosis or opportunistic infections. A purified protein derivative (PPD) skin test was not performed due to recent immunotherapy. The patient was referred to an infectious disease specialist for the evaluation of tuberculous lymphadenitis. Due to persistent fever and caseous granulomatous lymphadenitis findings, anti-TB treatment was initiated with isoniazid, ethambutol, rifampicin, and pyrazinamide. Concurrently, we continued nivolumab treatment for an additional 18 cycles. After one month of the anti-TB treatment, fever was controlled, and fatigue improved. After six months, the control F-18FDG PET/CT did not show any recurrence. Also, a complete response for melanoma was achieved with a total of 24 cycles of nivolumab. Anti-TB therapy was discontinued after 12 months. The patient was placed under medical surveillance, with follow-up examinations of 3 months intervals.

## 3. Discussion

In the recent past, oncolytic immunotherapy arose as a novel and promising approach for inhibiting tumor progression and metastasis [11]. ICIs mainly suppress uncontrolled immunity and reactivate tumor-specific T cells via suppressing checkpoint-mediated signals [12]. The immune-mediated effects of ICIs can lead to various types of IrAEs, and our knowledge and understanding of side effects are continually increasing with time. Although opportunistic infections are not expected side effects of ICIs, the diagnosis of ICI-related TB has been recently increasing. To date, a few cancer patients have been reported with activated TB during ICI therapy, however, to the best of our knowledge, there has been no tuberculous lymphadenitis case reported so far. In their systematic review of the literature, Zaemes et al. reported a total of 16 patients who developed active TB under PD-1 inhibitors therapy [9]. Ten patients were treated with nivolumab, and six of them were treated with pembrolizumab. The median age was 61, and 75% of the patients were male. Among all of the patients, lung cancer was the most common cancer, followed by melanoma. The median time of treatment initiation to TB reactivation was 6.3 months. Also, in their large cohort of 1144 South Korean solid cancer patients, Im et al. suggested that the inhibition of the PD-1 pathway is associated with the development of TB [13].

Active TB is generally developed in an immune-suppressed environment induced by cytotoxic chemotherapy or steroid administration [14]. The mechanism underlying the development of TB with PD-1 inhibitor use has not been elucidated yet. Triggering of excessive inflammatory responses with ICIs therapy is a potential cause. Preclinical studies with mice models have showed that immune checkpoint pathways have a crucial role in immune hemostasis in the reactivation of TB. They showed that PD-1 deficiency is related to worsening TB in mice models [15]. In their experimental animal studies, Barber et al. reported that PD-1 deficiency is related to excessive production of interferon-g (IFN-g) in their *Mycobacterium tuberculosis*-infected mice [16]. Additionally, over-inflammation and excessive necrosis was found in the lung tissue of PD-1 deficient mice [15]. This phenomenon is similar to the immune reconstitution inflammatory syndrome (IRIS) associated with the initiation of antiretroviral therapy in patients with AIDS [17]. Further studies are needed to better explain the underlying pathophysiological mechanism of TB after immune checkpoint inhibition.

Tuberculous lymphadenopathy is a local manifestation of systemic disease, known as the most common form of extrapulmonary TB. It can develop either during primary infection or reactivation of the latent lesions. Due to the low sensitivity of the mycobacterial culture in extrapulmonary TB, biopsy or surgery can provide definitive tissue diagnosis [6]. F-18FDG PET/CT scanning is a useful diagnostic modality in the diagnosis of malignant disorders and cancer screening. However, it is not only specific for cancer but also can be positive in granulomatous diseases [18]. Some previous cases reported positive F-18FDG PET/CT results in tuberculous lymphadenitis patients who had previous cancer history and were misdiagnosed as a recurrence of the malignancy [19,20]. In our case, cervical lymph nodes were first observed with increased FDG uptake in a PET/CT scan, and subsequently, tissue diagnosis showed tuberculous lymphadenitis without malignant cell involvement.

In the current literature, it is unclear whether ICI therapy can be continued after the development of active TB or when it is safe to resume ICIs. Among 16 previously reported cases, immunotherapy was stopped in all patients except one [21]. None of the patients who restarted immunotherapy had signs of recurrence or worsening of TB. In our case, we continued nivolumab treatment along with the anti-TB regimen. No recurrence or worsening of the TB was observed.

## 4. Conclusions

TB reactivation can occur in various organs as a complication of PD-1 targeted immunotherapy. Considering the increased utilization of ICI-based immunotherapies, this issue could cause significant mortality and morbidity, especially in populations with high TB prevalence. It is necessary to conduct further studies during ICI therapy in patients whose baseline TB status is known. We recommend TB screening in all patients before starting PD-1 inhibitor therapy.
